# The Guinea Pig as a Model for Sporadic Alzheimer’s Disease (AD): The Impact of Cholesterol Intake on Expression of AD-Related Genes

**DOI:** 10.1371/journal.pone.0066235

**Published:** 2013-06-21

**Authors:** Mathew J. Sharman, Seyyed H. Moussavi Nik, Mengqi M. Chen, Daniel Ong, Linda Wijaya, Simon M. Laws, Kevin Taddei, Morgan Newman, Michael Lardelli, Ralph N. Martins, Giuseppe Verdile

**Affiliations:** 1 Centre of Excellence for Alzheimer’s Disease Research and Care, School of Medical Sciences, Edith Cowan University, Perth, WA, Australia; 2 School of Human Life Sciences, University of Tasmania, Launceston, Tasmania, Australia; 3 Discipline of Genetics, School of Molecular and Biomedical Science, The University of Adelaide, Adelaide, SA, Australia; 4 Sir James McCusker Alzheimer’s Disease Research Unit, Hollywood Private Hospital, Nedlands, WA, Australia; 5 School of Psychiatry and Clinical Neurosciences, University of Western Australia, Crawley, WA, Australia; The University of Queensland, Australia

## Abstract

We investigated the guinea pig, *Cavia porcellus*, as a model for Alzheimer’s disease (AD), both in terms of the conservation of genes involved in AD and the regulatory responses of these to a known AD risk factor - high cholesterol intake. Unlike rats and mice, guinea pigs possess an Aβ peptide sequence identical to human Aβ. Consistent with the commonality between cardiovascular and AD risk factors in humans, we saw that a high cholesterol diet leads to up-regulation of BACE1 (β-secretase) transcription and down-regulation of ADAM10 (α-secretase) transcription which should increase release of Aβ from APP. Significantly, guinea pigs possess isoforms of AD-related genes found in humans but not present in mice or rats. For example, we discovered that the truncated PS2V isoform of human PSEN2, that is found at raised levels in AD brains and that increases γ-secretase activity and Aβ synthesis, is not uniquely human or aberrant as previously believed. We show that PS2V formation is up-regulated by hypoxia and a high-cholesterol diet while, consistent with observations in humans, Aβ concentrations are raised in some brain regions but not others. Also like humans, but unlike mice, the guinea pig gene encoding tau, *MAPT*, encodes isoforms with both three and four microtubule binding domains, and cholesterol alters the ratio of these isoforms. We conclude that AD-related genes are highly conserved and more similar to human than the rat or mouse. Guinea pigs represent a superior rodent model for analysis of the impact of dietary factors such as cholesterol on the regulation of AD-related genes.

## Introduction

Murine models of Alzheimer’s disease (AD) have played an important role in providing significant insight into mechanisms underlying disease pathogenesis and are still currently the most commonly used for pre-clinical drug screening. Rodent models themselves are poor natural models of AD and do not exhibit pathological hallmarks of the disease [deposition of beta amyloid (Aβ) and aggregation of tau as neurofibrillary tangles], partly due to differences in Aβ and tau species and aggregation states of these proteins. Thus, transgenic models, expressing familial AD (FAD) associated mutations in key components of Aβ metabolism [i.e. amyloid precursor protein- APP and/or presenilins (PS) genes) and tau *(MAPT)]* have been developed [reviewed in [1]]. The relevance of these models to the more common late onset AD (LOAD) which is associated with a complex aetiology, maybe questioned. Further, limitations of the murine models associated with transgene expression [2], differences in genetic background [3] and confounding issues with the presence of both human and endogenous murine Aβ and tau [4], has prompted growing interest in further investigating non-transgenic animal models, such as the guinea pig (*Cavia porcellus*).

To date, the presence of neurofibrillary tangles or compact senile plaques has not been reported in the guinea pig brain. A recent report has showed that the closely related *Octodon degus* exhibits an age dependent accumulation of these neuropathological markers of AD [5], suggesting that guinea pigs may show similar age related changes, however comprehensive ageing studies in guinea pig are lacking. Nevertheless, studies revealing that APP in guinea pig is highly conserved with that of humans and that the Aβ sequence is identical [6], [7] prompted the use of this rodent model in assessing amyloid lowering therapeutics [8], [9], [10], [11] and hormonal regulation of Aβ metabolism [12], [13], [14].

Guinea pigs are also an excellent non-transgenic animal model in which to study the mechanism underlying the effects of cardiovascular risk factors, nutrition and drug interventions on AD-like pathology as they are the only small animal model that closely mimics human lipoprotein and cholesterol metabolism [15]. In contrast to other rodents and most species used for studying lipid metabolism, guinea pigs carry the majority of their plasma cholesterol in LDL, the atherogenic lipoprotein, similar to humans making them a unique animal model with which to study cholesterol and lipoprotein metabolism [15]. They are also excellent models to evaluate dietary interventions as they show aortic plaque accumulation when challenged with a hypercholesterolemic diet [15], [16], [17].

The guinea pig has not been widely used to assess the impact of dietary interventions on AD related pathology, such as Aβ accumulation. One possible reason for the guinea pig not being widely used in such studies is that apart from APP and Aβ, the conservation of AD-related genes and their regulatory responses to major risk factors involved in AD, has not been thoroughly explored. Considering this we investigated whether certain AD genes, particularly those involved in APP and Aβ metabolism, are conserved in guinea pigs and we assessed the regulation of these genes under conditions of the major risk factor - high dietary cholesterol intake.

## Methods

### Ethics Statement

This study was carried out in strict accordance with the recommendations in the Australian code of practice for the care and use of animals for scientific purposes of the National Health and medical research Council (NHMR&C). The protocol was approved by the Committee on the Ethics of Animal Experiments of Edith Cowan University (Approval number 05-A17). The guinea pigs were anaesthetised with isoflurane prior to euthanasia. All efforts were made to minimise suffering.

### Sequence Alignments of AD-related Genes

To analyse the sequential similarity of AD-related genes in human and rodent models, blastp analysis was performed, using the NCBI blast engine (http://blast.ncbi.nlm.nih.gov/Blast.cgi?). Default parameters were used, with the exception that a gap existence penalty of 10 and a gap extension penalty of 1 were applied. The Sequence Similarity Score was calculated as shown at http://www.ncbi.nlm.nih.gov/BLAST/tutorial/Altschul-1.html and was the main parameter used to judge conservation between the human AD-related genes and their orthologues in rodent models.

### Animals

Sixteen male Hartley guinea pigs weighing 500 to 600 *g* were obtained from the Biological Sciences Animal Unit at the University of Western Australia (Perth, WA, Australia). At the beginning of the study, animals were randomly assigned to one of two experimental groups, a control diet, and a high-cholesterol diet for 12 weeks.

Guinea pigs were housed in a controlled environment at 22°C on a 12 hour day/night cycle (light from 0700 to 1900 h). Diet and water were consumed *ad libitum*. The guinea pigs were weighed before and during each week of the study to monitor their health. Compared to animals fed the control diet, no significant changes were observed in food consumption or body weight of animals fed the cholesterol diet (Figure S1).

For analysis of the response of PS2V to a hypoxia mimetic in guinea pig brains, brains from three culled adult guinea pigs were collected from the colony maintained by the Veterinary Services Division of IMVS Pathology in Adelaide. Brains were divided into left and right halves before cutting into small (≤1 mm diameter) pieces and incubation for 6 hours in either DMEM medium plus FCS or this medium containing 100 µM NaN_3_ followed by mRNA extraction and qPCR (see below). The same procedure was used to test for PS2V formation in an adult mouse brain collected from another research project at The University of Adelaide.

### Diets

The research diets were prepared and pelleted by Specialty Feeds (Glen Forrest, WA, Australia). The control diet consisted of 0.25% cholesterol, 34% fat, 25% protein and 41% carbohydrate (Table S1). This cholesterol diet has commonly been used in guinea pigs to cause hypercholesterolemia and induce atherosclerotic plaque accumulation [16], [17], [18], [19]. This amount of dietary cholesterol corresponds to an absorbed amount equal to 1.5 times the daily cholesterol synthesis rate in guinea pigs [18] and is the equivalent to 1,875 mg cholesterol per day in the human situation. The control diet used consisted of 0.01% cholesterol, 34% fat, 25% protein and 41% carbohydrate. Both the cholesterol and control diets contained the same macronutrient composition and differed only in the cholesterol content. The experimental diets were weighed daily to monitor food intake.

### Tissue Collection and Sample Preparation

Guinea pigs were euthanized under isoflurane vapours and blood was obtained via cardiac puncture. Serum and cerebrospinal fluid (CSF) samples were collected and stored at −80°C for subsequent analysis of CSF and serum cholesterol. Animals were transcardially perfused with phosphate-buffered saline (PBS) with heparin (10 IU/mL) and the brains collected and snap frozen in liquid nitrogen. The dissected brain sections for protein analysis were homogenized in 1∶3 in PBS, pH 7.4 containing protease inhibitor cocktail tablets (Roche Diagnostics, Castle Hill, NSW, Australia) as described previously [20]. Protein concentrations were determined using a bicinchoninic acid (BCA) protein assay kit (Pierce, Rockford, IL, USA).

### Measurement of Aβ by ELISA

A sensitive double-antibody sandwich ELISA was used for the detection and measurement of brain and CSF Aβ. Brain homogenates were diluted 1∶10 with tissue homogenisation buffer, pH 7.4 (250 mM sucrose, 20 mM Tris-HCl, 1 mM EDTA, 1 mM EGTA) and Aβ extracted from brain homogenates with 0.4% diethylamine (DEA), 100 mM NaCl [21]. CSF samples were also diluted 1∶10 with PBS, prior to analysis. The Aβ ELISA assay was performed as previously described by Mehta et al. [22]. Briefly, brain and CSF Aβ levels were measured in prepared samples (100 µL) using monoclonal antibody WO2 as the capture antibody, with rabbit antiserum R208 (specific for Aβ40), kindly provided by Dr. Pankaj Mehta (NYS Institute for Basic Research, Staten Island, NY, USA) used as the detection antibody.

### Cholesterol Analysis

Serum cholesterol concentrations were determined using the Amplex Red Cholesterol kit (Molecular Probes, Leiden, Netherlands). Serum samples were assayed in duplicate using black 96-well plates. Plates were incubated in the dark for 30 min at 37°C and read using a FLUOstar OPTIMA multi-detection microplate reader (BMG Labtech Inc, Offenburg, Germany) at an absorption/emission spectrum of 560 nm/615 nm. The cholesterol concentrations of the samples were calculated from a cholesterol standard curve.

### Quantitative RT-PCR Analysis of ADAM10, BACE1 and PS2V Transcripts

Total RNA was extracted from frozen Guinea pig brain tissues, using Qiagen RNeasy Lipid Tissue Mini Kit (Cat No. 74804). The quality of extracted total RNA was assessed on 1% agarose gel and the quantity was determined spectrophotometrically using the Nano-spectrum instrument (Thermo Fisher Scientific). First strand cDNA was synthesised following the production manual, using Bioline cDNA synthesize Kit (Cat No. Bio-65025), and was stored at −20°C for future PCR.

The relative standard curve method for quantification was used to determine the expression of experimental samples compared to a basis sample. For experimental samples, target quantity was determined from the standard curve and then compared to the basis sample to determine fold changes in expression. Gene specific primers were designed for amplification of target cDNA (Table 1) and the cDNA from the ubiquitously expressed control gene *RPS-16*. The reaction mixture consisted of 50 ng/*µ*l of cDNA, 18 *µ*M of forward and reverse primers and Power SYBR green master mix PCR solution (Applied Biosystems, Foster City, USA).

**Table 1:** Forward and reverse primers used for the qRT-PCR of *ADAM10*, *BACE1*, *PSEN2*, *PS2V* and *MAPT* transcripts.

Gene	Forward Primer	Reverse Primer
*RPS16*	5′-AGACAGCTACAGCCGTGGCACAT-3′	5′-CAGAAGCAGAACAGGCTCCAGTAACTT-3′
*ADAM10*	5′-GTGATCGCCCAGATATCCAGT-3′	5′-GAACCCCATCATCAAAGTCTCG-3′
*BACE1*	5′-GAGATCGCCAGGCTCTGTG-3′	5′-CCACGATGCTCTTGTCATAGTTG-3′
*PS2V* (RT-PCR)	5′-ACGGTCAGCTTCATCCAG-3′ (in *Psen2* exon 3)	5′-TCAGGAAGAGCGTGGGGTAA-3′ (in *Psen2* exon 7)
*PSEN2 (qPCR)*	5′-CCGCTGCTACAAGTTCATCCA-3′	5′-CCACGTTGTAGGTCTTGAGCACT-3′
*PS2V* (qPCR)	5′-GCTTTCATCCACGGCTG-3′ (spans *Psen2* exon 4/6 junction)	5′-CCGAGGTAGATGTAGGTGAAC-3′ (in *Psen2* exon 6)
*MAPT* (RT-PCR for 3R and 4R)	(5′-ACTCCACCCAAATCACCCTCCTC-3′)	(5′-TTGATGCTGCCAGTGGAAGAGAC-3′)
*MAPT* (RT-PCR for 0N, 1N and 2N)	(5′- TTCTCCTCCACTGTCCTCTTCTG-3′)	(5′- GTGTCTCCAATGCCTGCTTCTTC-3′)
*MAPT (qPCR for full length)*	5′-TCCACCGAGAACCTGAAGCA-3′	5′-GATGTTGCCTAGCGAGCGG-3′
*MAPT (qPCR for 3R)*	5′-GGAAGGTGCAAATAGTCTACAAACC-3′	5′-CGCTCGCTAGGCAACATCTC-3′
*MAPT* (qPCR for 4R)	5′-TAGCAACGTCCAGTCCAAGTGT-3′	5′-CGCTCGCTAGGCAACATCTC-3′

Note that guinea pig exon designations are according to the cognate exons in human since annotation of the guinea pig genome sequence is currently rudimentary.

To generate the standard curve cDNA was serially diluted (100 ng, 50 ng, 25 ng, 12.5 ng). Each sample and standard curve reaction was performed in triplicate for the *RPS-16* gene and experimental genes. Amplification conditions were 2 min at 50 °C followed by 10 min at 95 °C and then 40–45 cycles of 15 s at 95 °C and 1 min at 60 °C. Amplification was performed on an ABI 7000 Sequence Detection System (Applied Biosystems, Foster city, USA) using 96 well plates. Cycle thresholds obtained from each triplicate were averaged and normalized against the expression of *RPS-16.* Each experimental sample was then compared to the basis sample to determine fold changes of expression. Each experiment was conducted three times and triplicate PCRs were performed for each sample.

### Sequence Prediction of Guinea pig Mapt and Subsequent RT-PCR Analysis

To predict the full sequence of guinea pig Mapt, tblastn analysis against guinea pig ESTs and genomes was performed, using human Mapt protein sequence as a query. The resulting fragments were then assembled based on overlapping sequence and their genomic location guided by information in the guinea pig Ensembl genome browser (http://www.ensembl.org/). RT-PCR was performed to identify 3R and 4R or 0N, 1N and 2N transcripts of *Mapt* using primer pairs outlined in table 1. The PCR products were then examined on 1% agarose gel and sequenced.

### Statistical Analyses

Means and standard deviations were calculated for all variables using conventional methods. A students t-test was used to evaluate significant differences among the two groups of animals for the Aβ levels in the CSF and brain, levels of BACE1, ADAM10 and, PSEN2 and PS2V transcripts and serum cholesterol levels. Raw *p* values, degrees of freedom and *t* values are shown within the figure and figure legend. All values represent mean ± SEM of 8 animals per group. A criterion alpha level of P<0.05 was used for all statistical comparisons. All data were analysed using SPSS version 15.0 (SPSS, Chicago,IL).

## Results

### Sequence Similarities of AD-related Genes between Human, Mice Rats and Guinea Pig

**Figure 1:**
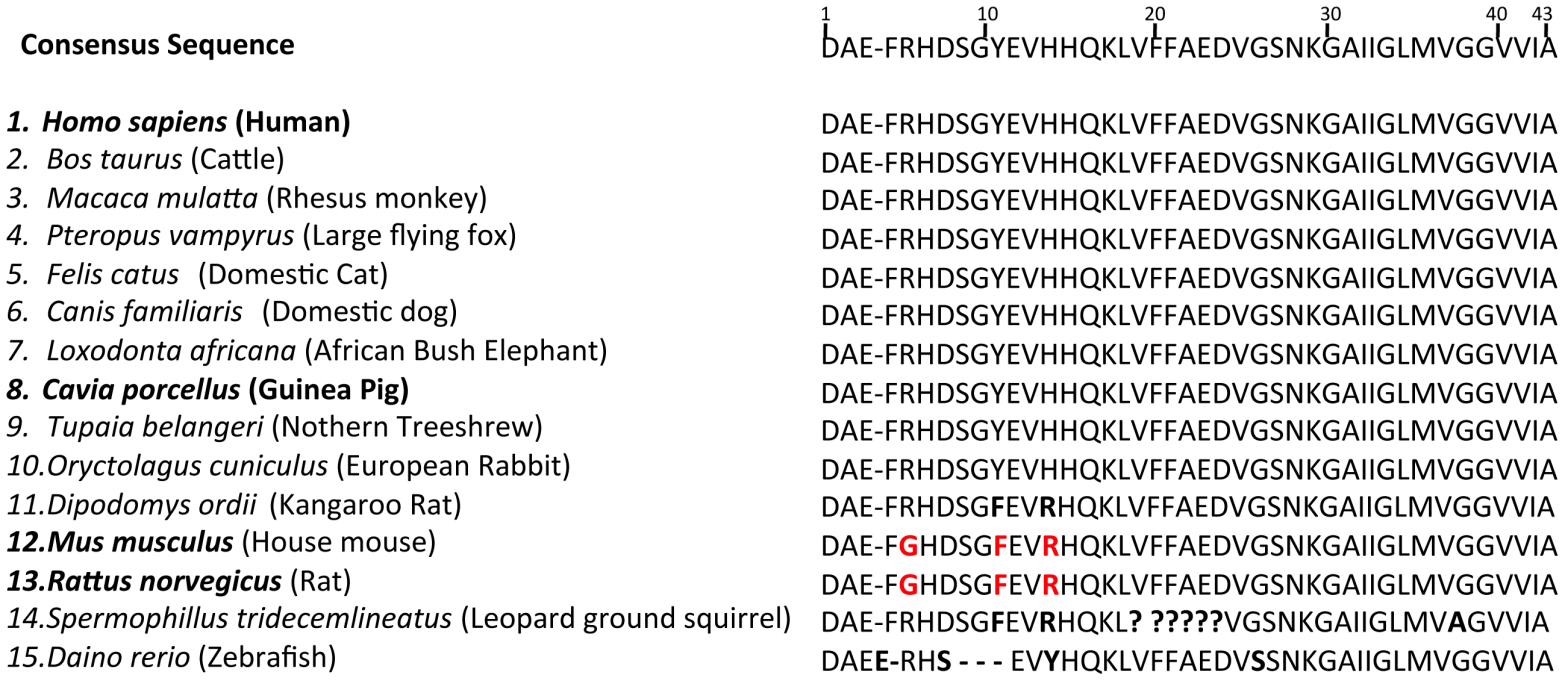
Amino acid residue sequence alignment of Aβ in humans and that predicted for guinea pig, rat and mouse. Black shading indicates identical residues. Red box represents residues from mouse and rat Aβ that differ from those in human and guinea pig Aβ sequences.

Although, the sequence identity of guinea pig Aβ to that of human has been well established [(Figure 1, [7], [6])], genetic similarities with other AD related proteins have not been well documented. Therefore, we investigated the similarities of AD-related proteins between common rodent models (i.e. guinea pigs, mice and rats) and humans. These included, the Aβ parent molecule, APP and its processing enzymes, β-APP CLEAVING ENZYME 1 (BACE1) and A DISINTERGRIN AND METALLOPROTEINASE 10 (ADAM10); two critical components of the mutli-subunit γ-secretase enzyme, PRESENILIN1 (PSEN1), and PRESENILIN2 (PSEN2), Aβ clearance proteins APOLIPOPROTEN E (APOE) and INSULIN DEGRADING ENZYME (IDE) and the component of neurofibrillary tangles, MICROTUBULE ASSOCIATED PROTEIN TAU (MAPT). For each protein, a “pblast” test was performed to compare sequence similarities between guinea pigs, mice or rats and humans. The “Sequence Similarity Score” was used as the main parameter to determine the level of sequence similarity. Results from the analysis are shown in Table 2. As expected, all three rodents mostly show very similar levels of sequence similarity of AD-related genes to their human orthologues (See Table 2). However, this is not the case for Psen1 where guinea pig Psen1 shows 96% identity to human PSEN1 but the mouse and rat proteins show 92% and 93%, respectively. PSEN1 is the major FAD locus in humans and over 200 mutations are known to affect different amino acid residues (aa). For this reason up to a 4% difference in sequence identity for this 467 aa protein may well be significant in terms of function.

**Table 2:** Sequence Similarity Comparison of AD-related proteins, between Humans and species of rodent: Sequence Similarity Scores and Sequence Identity (%) shown for each gene.

	APP	PSEN1	PSEN2	BACE1	ADAM10	APOE	IDE	MAPT*
Guinea Pig	1269 (97%)	759 (96%)	761 (96%)	1001 (97%)	1316 (96%)	416 (70%)	1880 (97%)	655 (90%)
Mouse	1261 (97%)	740 (93%)	764 (96%)	1009 (96%)	1308 (96%)	442 (71%)	1848 (96%)	652 (89%)
Rat	1269 (97%)	743 (92%)	745 (95%)	1010 (96%)	1313 (96%)	410 (70%)	1849 (96%)	660 (90%)

* The MAPT sequence of guinea pig was predicted.

**Figure 2:**
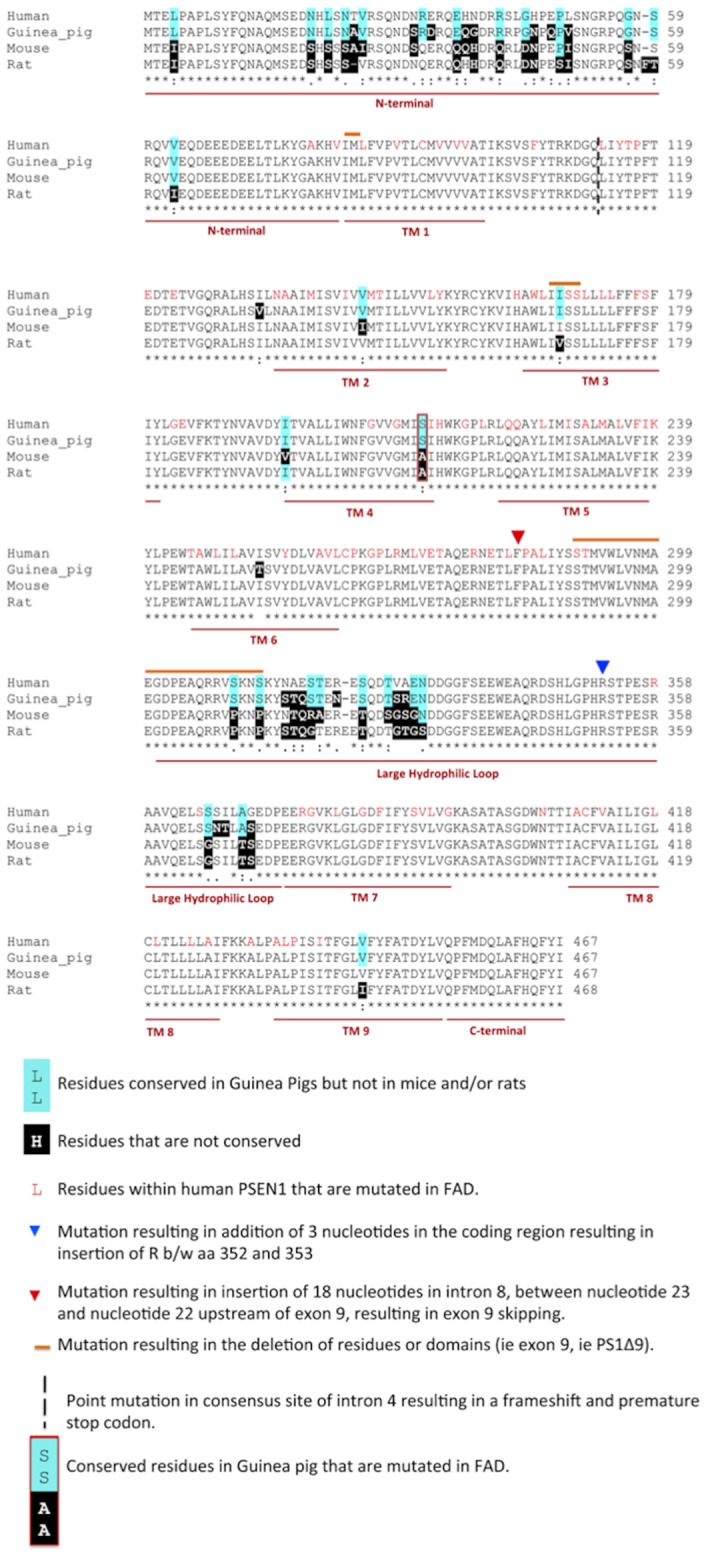
Amino acid residue sequence alignment of human PSEN1 and that predicted for guinea pig, rat and mouse. Residues that are conserved in human and guinea pig but not in the rat, mouse or both are shaded in blue. Rodent residues not conserved in humans are shaded in black. Residues known to be mutated in FAD in human PSEN1 are shown in red text. Only one residue is conserved in guniea pigs (but not mice and/or rats) that is mutated in FAD (S212Y).

Alignment of the PSEN1 protein sequence of rat, mouse and guinea pig to that of human PSEN1 (Figure 2) reveals a number of residues throughout the protein that are conserved in guinea pigs but not rats or mice. These residues appear to be concentrated within the N-terminus and large hydrophilic loop. Of the 100 residues in the PSEN1 protein in which FAD associated missense mutations occur, only one residue (serine) is conserved in guinea pigs but not conserved in, mice or rats. The mutation affecting this residue, S212Y, occurs in transmembrane 4 and has recently been identified in a family with FAD and shown to be associated with increased brain amyloid load, brain hypometabolism and increased Aβ42 production [23].

### The PS2V Marker of AD Pathogenesis is not Unique to Humans

The analysis of overall sequence similarity shown in Table 2 can conceal important differences in isoform formation generated by alternative splicing. An example of this is the splice donor sites present in exon 3 of human PSEN1 which result in variants that differ in a four amino acid (VRSQ) motif [24]. The presence or absence of this motif at the 3′ end of exon 3 affects the binding of the GDP dissociation inhibitor that recycle rab GTPases important for vesicle trafficking [25]. The donor splice site is not conserved in mice, resulting in the inability of the motif to be alternatively spliced leading to only the longer isoform of PS1. The imbalance of longer to shorter PSEN1 isoforms has been speculated to lead to differences in Aβ production [25].

**Figure 3:**
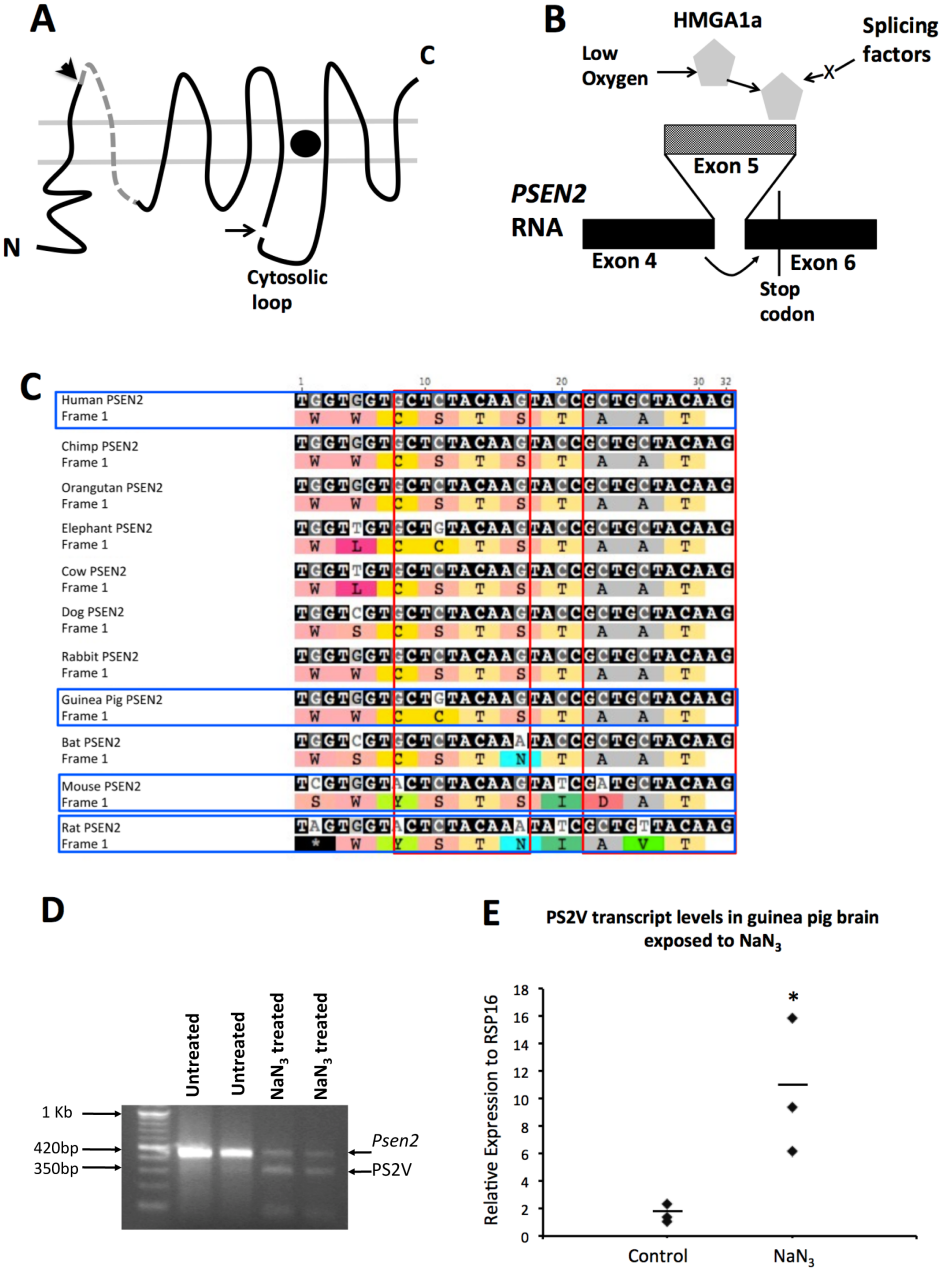
Formation of the PS2V Transcript. **A)** Presenilin structure in lipid bilayers: Arrowhead indicates boundary between protein sequences derived from exon 4 and 5. Dashed line indicates sequence from exon 5. Arrow indicates endoproteolysis site. Filled circle indicates γ-secretase catalytic site. **B)** PS2V forms when HMGA1a is expressed and binds to exon 5 (lighter shading) of *PSEN2* RNA causing ligation of exon 4 to exon 6 and ORF termination. **C)** Nucleotide sequence alignment of the 3′ end of exon 5 in human *PSEN2* RNA (with corresponding encoded residues) and the cognate exon of other species. Red boxes enclose sequences aligned with the HMGA1a-binding sites in human *PSEN2* RNA. **D)** mRNA from guinea brains exposed to control media or to media containing NaN_3_ followed by RT-PCR analysis using primers amplifying cDNA spanning exons 3 to 7 of *Psen2*. In untreated samples a prominent ∼420 bp band is observed. In NaN_3_ treated samples an additional ∼350 bp band is evident representing the cDNA fragment predicted from exclusion of the exon 5 sequence (PS2V). **E)** qPCR using a primer spanning the exon 4/6 junction PS2V cDNA showed up-regulation of PS2V mRNA in samples treated with NaN_3_.

This prompted us to investigate whether there are species differences in isoforms of PSEN2, resulting from alternative splicing. A normal truncated PSEN2 isoform “PS2V” was identified by Sato and colleagues [26] and has implications in AD, since it shows increased expression in AD brains and up-regulates Aβ production [27], [28]. Human neuronal cells under oxidative stress induce expression of the HIGH MOBILITY GROUP AT-HOOK 1 (HMGA1) protein [29], [30]. This binds to specific sites within exon 5 of human *PSEN2* transcripts leading to exclusion of exon 5 and ligation of exon 4 and exon 6 sequences. The ligation of exon 4 to exon 6 sequences results in a frameshift that terminates the open reading frame in exon 6 and results in translation of a truncated PSEN2 protein isoform named PS2V (Figure 3 A and B).

Sequence alignment analysis of the HMGA1 binding site on PSEN2 in human, mouse, rat and guinea pig and other mammals revealed that this sequence is completely conserved in guinea pigs but not conserved in mice and rodents (Figure 3). Consistent with this, we were unable to detect PS2V transcript formation in PC12 (rat pheochromocytoma) cells and mouse brain following treatment with NaN_3_ to mimic hypoxia (see Materials and Methods, data not shown), supporting that HMGA1a could not bind to the *PSEN2* transcripts of these rodents to cause alternative splicing. To test for PS2V formation in guinea pigs we extracted mRNA from guinea brains exposed to NaN_3_ and then RT-PCR was conducted using primers amplifying cDNA spanning exons 3 and 7 of *Psen2*. This revealed the presence of a smaller cDNA fragment predicted from exclusion of exon 5 sequence (Figure 3D). qPCR using a primer binding over the exon 4/6 junction (and so amplifying only PS2V cDNA) showed that hypoxia mimicry significantly increases PS2V transcript levels (Figure 3E).

### PS2V is Up-regulated by the AD Risk Factor Cholesterol Intake

**Figure 4:**
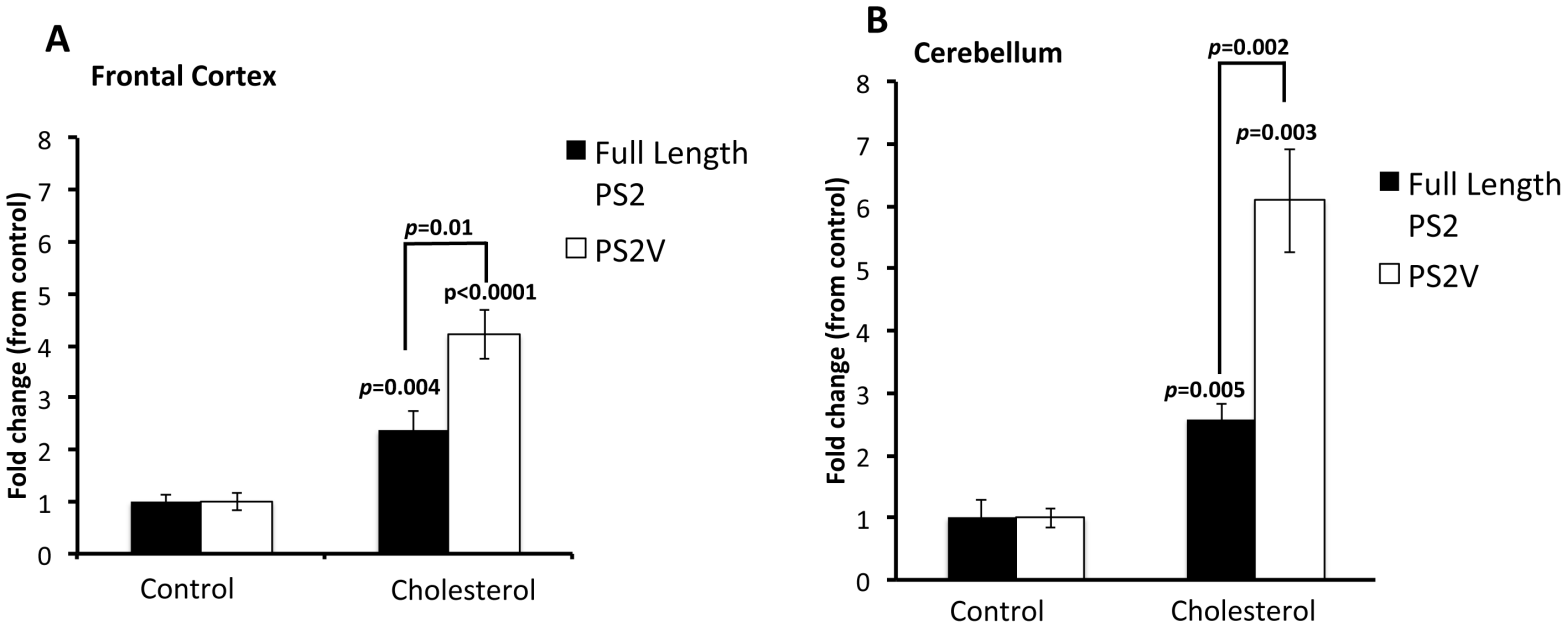
PS2V is up-regulated under cholesterol-fed conditions. Quantitative PCR analysis shows that, in comparison to animals fed a control diet, guinea pigs fed a cholesterol rich diet showed a significant increase in *PSEN2* and *PS2V* transcripts in (**A**) frontal cortex (*p* = 0.004; t = 3.429, d.f. = 14 and *p*<0.0001, t = 6.841, d.f. = 14, respectively) and (**B**) cerebellum (*p*<0.005; *t* = 4.484 and *d.f.* = 14 and *p*<0.003, t = 4.763, d.f. = 14, respectively). The fold increase of PS2V levels in these regions was greater than the increase in full length *PSEN2* levels [4 fold vs 2 fold in frontal cortex (*p* = 0.01, *t* = 2.994, *d.f.* = 14], and 6 fold vs 2 fold in the cerebellum (*p* = 0.002, *t* = 3.733, *d.f.* = 14)]. Data is represented as fold change from control fed animals. Transcript levels were normalised against RPS16. Data represents ± SEM.

Unlike rats and mice, guinea pigs metabolise cholesterol in very similar manner to humans. Since high cholesterol intake is a risk factor for AD and guinea pigs possess the AD marker PS2V, we examined whether PS2V levels are affected in the presence of this risk factor. Guinea pigs were fed a normal diet or a cholesterol diet for 12 weeks. Serum cholesterol concentrations were significantly increased in the cholesterol group compared to the control group (7.1±4.9 vs. 3.6±1.4 mMol/L respectively, p = 0.0017) at the completion of the 12 week intervention, confirming the effect of the cholesterol diet. To examine relative full length *PSEN2* and PS2V levels we then extracted mRNA from the frontal cortex and cerebellum for synthesis of cDNA followed by qPCR (Figure 4 A and B). Compared to control, full length *PSEN2* transcript levels increased ∼2 fold in both regions. The increase in PSEN2 expression is consistent with previous findings where human neuroblastoma cells were exposed to LDL-cholesterol [31]. However, we show dramatic increases in PS2V levels were observed where levels increased 4 fold and 6 fold in the frontal cortex (A) and cerebellum (B), respectively compared to control fed animals. The impact of cholesterol on PS2V levels was above that seen for *PSEN2* levels (p<0.01, and p<0.001 compared to *PSEN2* levels in cholesterol fed animals).

### Aβ and Genes Involved in Aβ Synthesis are Up-regulated by High Cholesterol Intake

**Figure 5:**
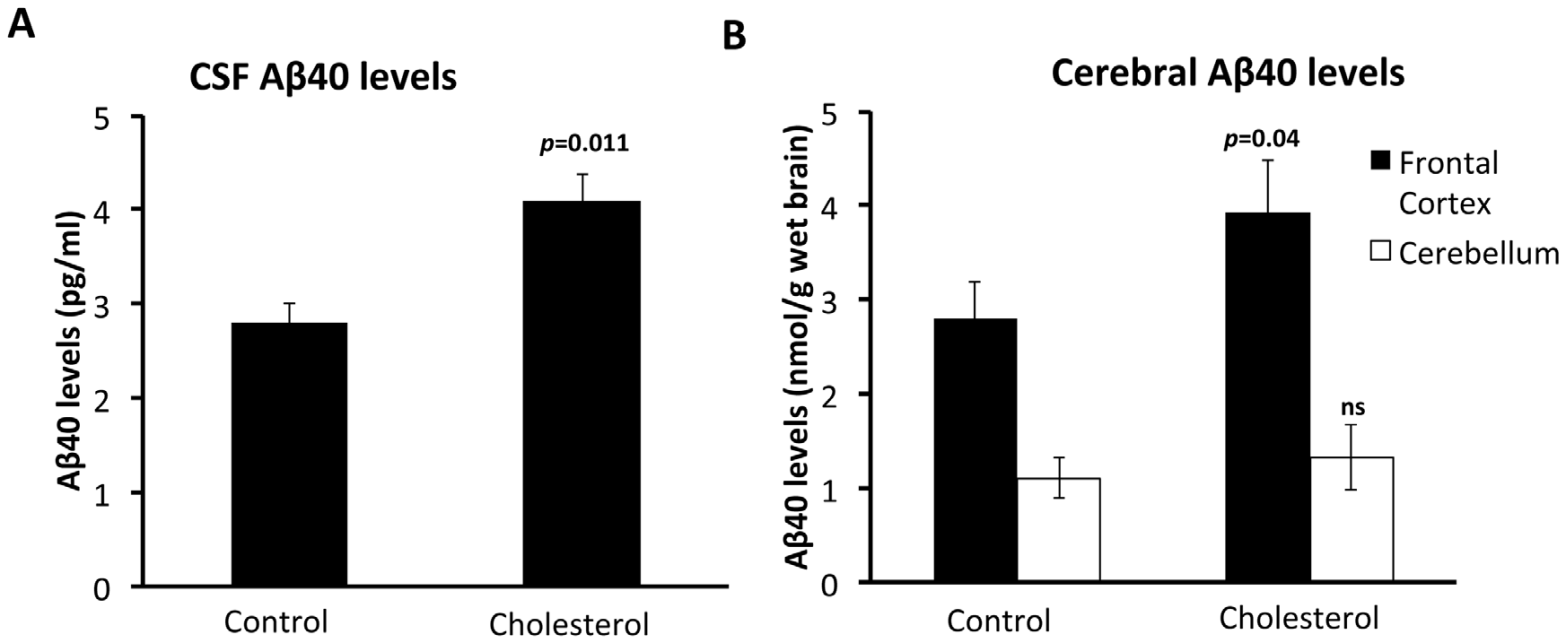
Increased Aβ1-40 levels in the CNS of cholesterol fed guinea pigs. **(A)** CSF Aβ1-40 levels (pg/mL) in the cholesterol and control fed diet groups following 12 weeks of feeding. Value is significantly increased over those animals fed the control diet (*p* = 0.011, *t* = 2.896, *d.f.* = 14). **(B)** Cerebral Aβ1-40 levels (nmol/g wet tissue) in frontal cortex and cerebellum homogenates from animals fed for 12 weeks on a high cholesterol or control diet. Increases are observed in animals fed cholesterol diet in the frontal cortex (*p* = 0.04, *t* = 2.204, *d.f.* = 14) but not in the cerebellum (*p* = 0.501, *t* = 0.684, *d.f.* = 14, ns). Values mean ± SEM.

**Figure 6:**
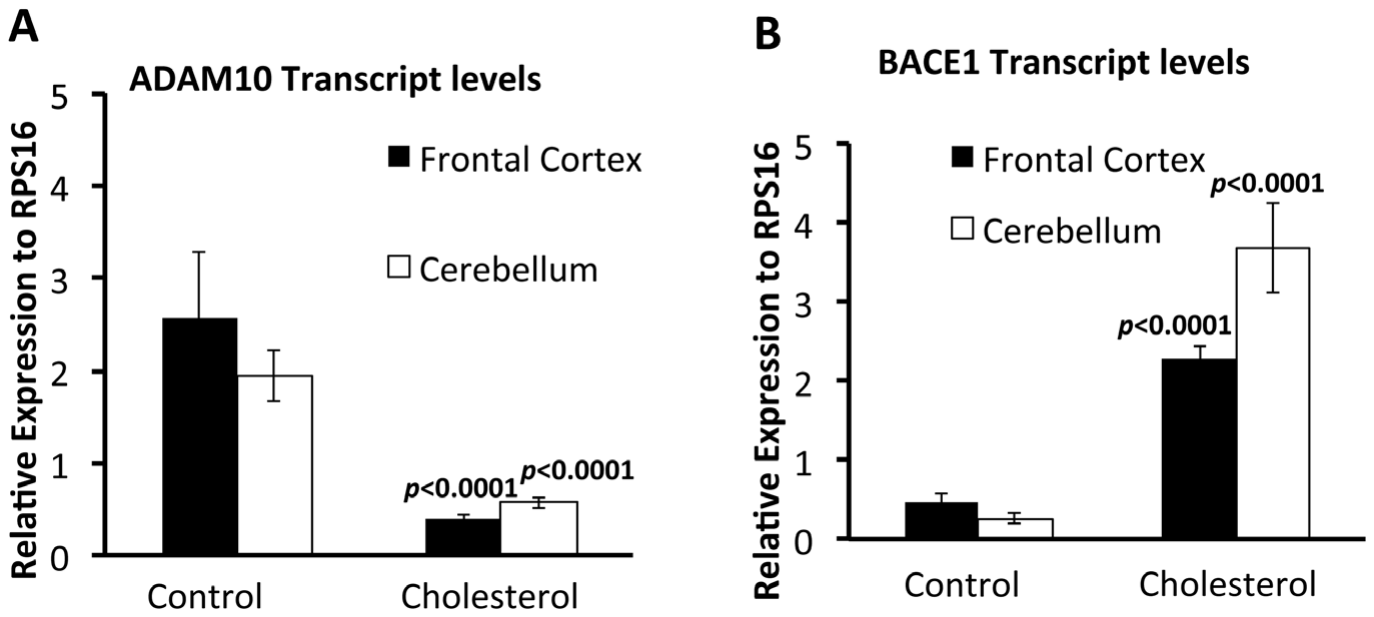
Increased *BACE1* RNA and reduced *ADAM10* RNA expression levels in brain tissue from guinea pigs fed a high cholesterol diet. Quantitative PCR analysis analysis for (A) ADAM10 and (B) BACE1 expression on total RNA extracted from the frontal cortex and cerebellum of guinea pigs fed the control or cholesterol diets. Data is represented as relative expression to RPS16. Compared to animals fed the control diet, ADAM10 expression is significantly decreased in the frontal cortex (*p*<0.0001, *t* = 7.735, *d.f.* = 14) and cerebellum (*p*<0.0001, *t* = 6.30, *d.f.* = 14) from animals fed cholesterol. In contrast BACE1 levels are significantly increased in the frontal cortex (*p*<0.0001, *t* = 8.196, *d.f.* = 14) and cerebellum (*p*<0.0001, *t* = 8.196, *d.f.* = 14). Values represent ± SEM.

Forced expression of PS2V in neuroblastoma cells increases γ-secretase activity and cleavage of Aβ from APP [27]. Also, increases in dietary cholesterol are known to correlate with higher Aβ cerebral load and changes in the APP processing enzymes BACE1 and ADAM10 [32], [33], [34], [35], [36]. Therefore, we tested whether increased cholesterol intake also affects the levels of Aβ, ADAM10 and BACE1 in guinea pig brains. Aβ was assessed by measuring levels in the cerebrospinal fluid (CSF), frontal cortex and cerebellum. Analysis of CSF Aβ40 levels showed a significant increase in Aβ40 in the cholesterol group compared to the control group (Figure 5A). Analysis of cerebral Aβ40 levels showed a significant increase in the frontal cortex for the HC group compared to the control group. There were no differences observed in the cerebellum between groups (Figure 5B). Quantitative RT-PCR (qRT-PCR) was used to assess BACE1 or ADAM10 expression levels. Results show that BACE1 transcript levels were significantly increased (Figure 6 A, B) and ADAM10 levels significantly reduced (Figure 6 C, D) in frontal cortex and cerebellum from guinea pigs fed the HC diet compared to those fed the control diet.

Overall, our findings indicate that cholesterol supplementation to guinea pigs up-regulates, PS2V, BACE1 and down-regulates ADAM10 expression, consistent with promoting Aβ production.

### Analysis of Mapt Transcripts in Guinea Pig Brain

**Figure 7:**
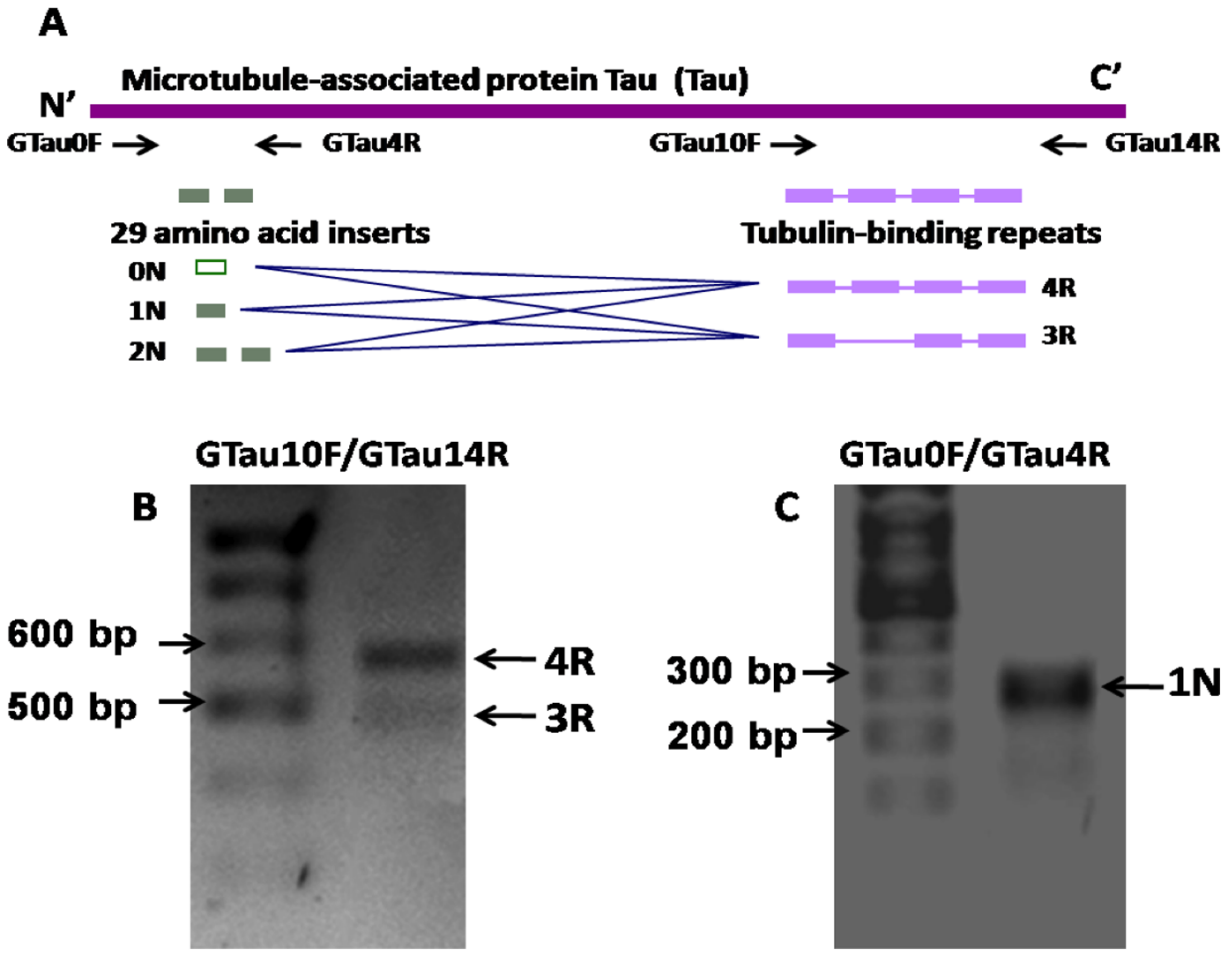
Mapt isoforms in guinea pig brain. **A)** Schematic diagram of the alternative splicing pattern of human *Mapt*. Six Mapt isoforms (0N3R, 1N3R, 2N3R, 0N4R, 1N4R, 2N4R) are generated from alternative splicing of exon2, 3 and 10 of the solo Mapt gene. The alternative splicing of exon2 or/and 3 (green boxes) yields *Mapt* isoforms with 0, 1 or 2 inserts of 29 amino acid residues in the N-termini; whereas, alternative splicing of exon 10 (purple) generates isoforms with either 3 or 4 tubulin-binding repeats in the C-termini. To analyse whether this splicing pattern is conserved in guinea pig, two primer pairs, GTau0F/4R and GTau10F/14R were designed, targeting the corresponding region of the human exon2/3 and tubulin-binding repeats domains respectively in guinea pig Mapt. **B)** RT-PCR *Mapt*, using primer pairs GTau10F/14R.c DNA was isolated from a brain sample from guinea pig fed normal chow diet Two bands representing 3R and 4R Mapt were detected. **C)** RT-PCR of Guinea pig *Mapt*, using primer pairs GTau0F/4R. A single band representing 1N *Mapt* was detected.

Although NFTs are also present in other dementias, they are still an important correlate of AD pathology and tau *(MAPT)* is a component of a toxic triad thought to mediate Aβ neurotoxicity [37], [38]. The strict regulation of *Mapt* transcriptional splicing, especially the maintenance of a 1∶1 ratio of the 3R and 4R isoforms (derived from the alternative splicing of Exon10 of the human *Mapt*) has been considered to play an important role in normal MAPT function. Disturbance of the 3R/4R ratio of MAPT has been evident in neurodegenerative diseases such as Frontotemporal dementia (FTD), Corticobasal degeneration (CBD), Progressive supranuclear palsy (PSP) and AD. In human brain, six MAPT isoforms are generated through alternative splicing of Exon 2, 3 and 10 (Figure 7A). The alternative splicing of exon 10 yields two groups of MAPT isoforms with either 3 or 4 microtubule-associate repeats on the C-termini of the protein. Alternative splicing of exon 2 and 3 yields Mapt isoforms with 0 (0N), 29(1N) or 58 (2N) amino acids. Mapt expression in mouse is notable for its lack of an isoform with 4 tubulin-binding repeats (4R) indicating that simple protein aa identity may be a poor indicator of conservation of protein function. Therefore, we sought to analyse the isoforms that could be produced by the guinea pig *Mapt* gene.

The number of tau *(MAPT)* isoforms present in guinea pig brain has not been widely investigated, most likely due to the full sequence of the Guinea Pig Mapt yet to be determined. For sequence analysis we used the predicted sequence of Guinea pig Mapt based on protein sequence alignments, using Guinea pig ESTs (http://blast.ncbi.nlm.nih.gov) and the Ensembl database (http://www.ensembl.org/) and Genome sequence database. As this is a predicted sequence, the true similarity scores may not be accurately reflected. The predicted guinea pig *MAPT* sequence shows a similar degree of identity to human *MAPT* as do those of the other rodents (Table 1). We investigated the presence of *Mapt* transcripts in guinea pig brain by RT-PCR. Two primer pairs were, Gtau0F/4R and Gtau10F/14R were designed, targeting the corresponding region of human exon 2 and 3 and the tubulin-binding repeats domains in Guinea Pig *Mapt*, respectively (see Figure 7A). Using these primers in RT-PCR of mRNA isolated from guinea pig brain, we observed the presence ∼600 bp and 500 bp transcripts corresponding to 3R and 4R repeats (Figure 7B) and a single transcript at ∼300 bp, corresponding to the 1N isoform.

**Figure 8:**
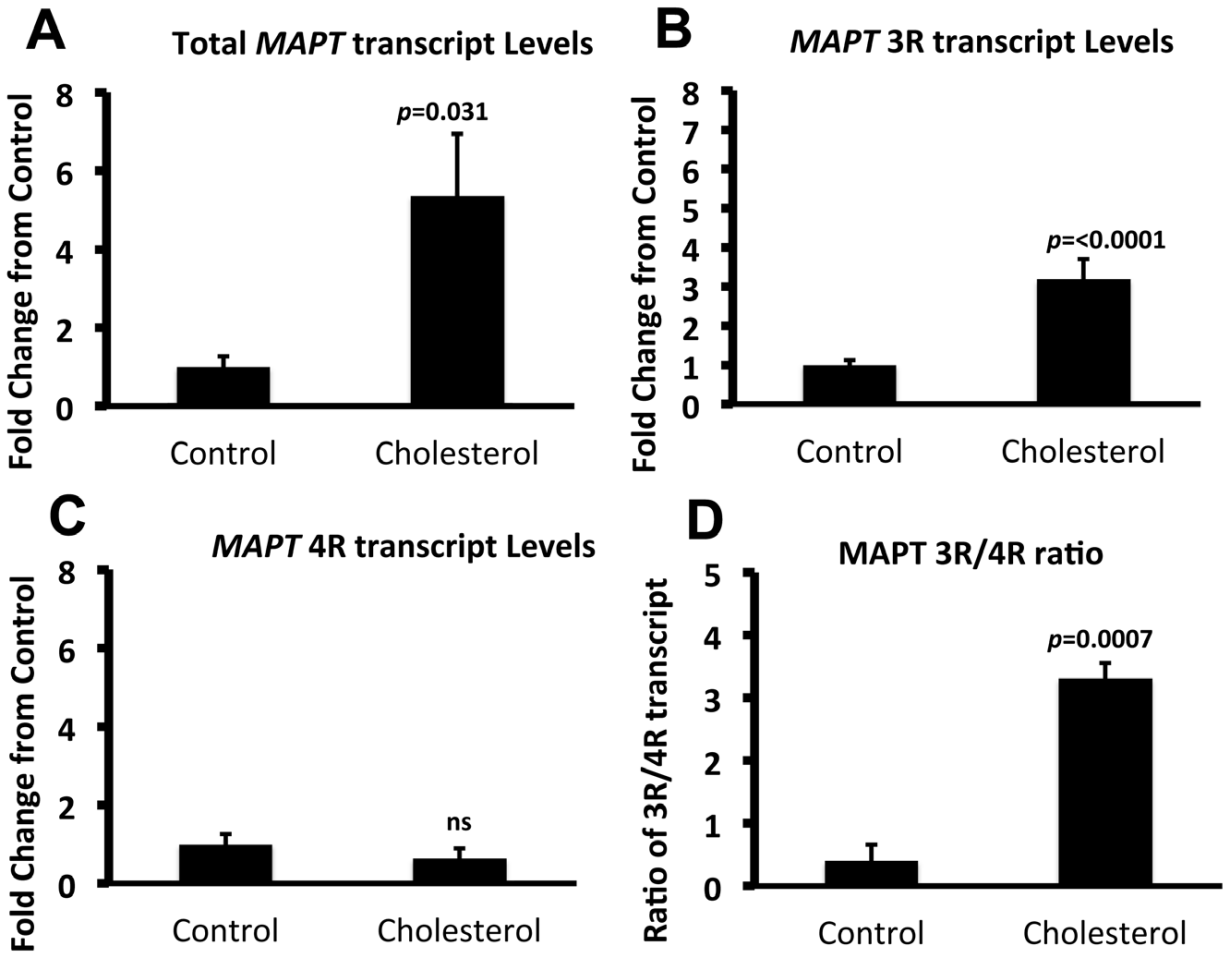
Total *MAPT* and *MAPT*3R transcripts are up-regulated under cholesterol fed conditions. Quantitative PCR analysis shows that, in comparison to animals fed a control diet, guinea pigs fed a cholesterol rich diet showed a significant increase in (A) total *MAPT* (*p* = 0.031, *t* = 3.560, *d.f.* = 14) and (B) *MAPT3*R (*p*<0.0001, *t* = 6.468, *d.f.* = 14) transcripts but (C) no change was observed in *MAPT4R* transcripts (*p* = 0.1320, *t* = 1.60, *d.f.* = 14, ns). An increased 3R/4R ratio was observed (*p* = 0.0007, *t* = 4.326, *d.f.* = 14). Data is represented as fold change from control fed animals. Transcript levels were normalised against RPS16. Data represents ± SEM.

Having identified the presence of *MAPT* transcripts in guinea pig brain, the impact of cholesterol on total, 3R or 4R *MAPT* transcripts was assessed. Quantitative PCR analysis of frontal cortex, revealed ∼5 fold increase in total MAPT levels in cholesterol fed guinea pigs, compared to animals fed a normal diet (Figure 8A). Transcript levels of MAPT3R significantly increased (Figure 8B), whilst no change was observed for MAPT4R transcripts (Figure 8C), resulting in an increase in the 3R/4R ratio (Figure 8D). Overall, the results show that although guinea pigs do not contain all isoforms of MAPT, unlike mice, [39], they contain 3R MAPT transcript which is up-regulated under cholesterol fed conditions.

## Discussion

### Guinea Pigs Show Closer Sequence and Isoform Similarity of AD Genes to Humans, than do mice and Rats

In the current study, in addition to comparing the sequence homology of APP and Aβ in human and the rodent species, mice, rats and guinea pigs, analysis was extended to other AD genes or genes that have been implicated in Aβ metabolism and clearance. All three rodents showed very similar levels of sequence similarity of APP to their human orthologue (97%). Analysis of IDE, ADAM10 and BACE1 also showed similar levels of sequence identity (96/97%), whereas APOE showed only 70% identity. Analysis of PSEN1 revealed that guinea pig PSEN1 showed 96% identity to human PSEN1 but the mouse and rat proteins showed 92% or 93%, respectively. The up to 4% difference in sequence identity between human and rodent may have significant implications in presenilins function and AD related neurodegeneration. This is highlighted by a recent study that analysed the human and mouse brain transcriptome and identified significant differences in transcriptional patterns in AD related genes between human and rodents [40]. Of particular note in that study was that PSEN1 was highly correlated with oligodendrocyte markers only in human brain tissue [40]. Oligodendrocytes are important in axon myelination, where a dysfunction of these cells leads to disruptions in neuronal communication network and neuronal degeneration. This close association of human PSEN1 with oligodendrocyte function may help to explain, in part, significant differences in neurodegeneration observed in human AD brain compared to those observed in mouse models.

A comparison of human PSEN1 sequence with that of rat, mice and guinea pig showed that this sequence divergence was mainly within the N-terminus and the hydrophilic loop. The residues within the transmembrane domains remain relatively conserved amongst guinea pigs, rats and mice. This is not surprising as these domains have been shown to be important in γ-secretase activity [41], [42], [43], [44], [45]. However, the N-terminal domain and the hydrophilic loop also exhibit important functions. The large hydrophilic loop has been shown to differentially regulate γ-secretase activity on APP and Notch [46] and is also important for γ-secretase-independent functions of the presenilins by interacting with proteins involved in intracellular trafficking [Rab11, [47]], cell-cell adhesion [48], anchoring of membrane proteins to the cytoskeleton [actin-binding protein 280, [49]] and synaptic activity [syntaxin 1A, [50]]. The N-terminal domain has been shown to be important in the formation of PS1 isoforms as a result of alternative splicing, which can impact on activity [25]. The interactions and activities of these domains and the formation of alternative protein isoforms are most likely to be conserved in those species showing greater sequence identity.

In contrast to PSEN1, analysis of PSEN2 revealed similar levels of sequence identity between human PSEN2 and the PSEN2 genes of mice, rats and guinea pigs. For the first time we demonstrate the presence of transcripts of the PS2V isoform in the guinea pig brain. As discussed below, this has important implications in AD as evidence is mounting that PS2V may play an important role in modulating Aβ metabolism under conditions of hypoxia/oxidative stress.

### Guinea Pigs, a more Suitable Small Animal Modelling the Impact of Cholesterol Loading on AD Related Proteins

Studies utilising animal models of AD, including rabbits [32], [33] and transgenic mice, [34], [35], [51] have all shown a strong correlation between serum cholesterol levels and cerebral Aβ production. Our results demonstrate a similar correlation in guinea pig brain. We showed that, in guinea pigs, cholesterol up-regulates BACE1 and down-regulates ADAM10 expression, which would contribute to the promotion of amyloidogenic processing of APP to generate Aβ. This mirrors previous findings where modulating cholesterol (either through supplementation or depletion) alters the expression BACE1 and ADAM10 *in vitro* [52] and *in vivo* in rat, transgenic mice, dog and rabbit models [32], [33], [34], [35], [53], [36]. This further establishes the suitability of guinea pig as an alternative model to undertake such dietary intervention studies.

Interestingly, the expression profile of PS2V, BACE1 and ADAM10 did not correlate with Aβ40 levels observed in the cerebellum of cholesterol fed animals. Increases in dietary cholesterol have been shown previously in rabbits to increase Aβ levels in the frontal cortex but not in cerebellum [54]. Although APP processing enzymes are expressed in the cortical and limbic areas that develop significant Aβ deposition, high expression is also seen in the cerebellum [55], which does not exhibit significant Aβ pathology. A number of studies have shown that expression of these enzymes is not related to age or regional neuritic plaque burden [55], [56], [57], [58] and suggest that other factors such as Aβ catabolism/clearance may influence the accumulation of Aβ in certain brain regions.

Guinea pigs are the only small animal model in which generation of PS2V has been identified. The PS2V transcript was previously observed in human neuroblastoma cells under conditions of hypoxia-generated oxidative stress and in the brains of individuals with sporadic, late onset AD [27], [28], [59]. Over-expression of PS2V up-regulates Aβ production in neuroblastoma cells [27]. Our results show, for the first time, that an additional stimulus, hypercholesterolemia, simulates PS2V production in addition to up-regulating Aβ synthesis.

The up-regulation of PS2V could be a contributing factor modulating Aβ in hypercholesterolemia. Hypercholesterolemia can lead to vessel wall changes in the brain, leading to hypoperfusion, ischemia and hypoxia [reviewed in ([60]] and evidence indicates that this can contribute to AD pathogenesis. Hypoxia induced by cerebrovascular hypoperfusion in rats lead to accumulation of cerebral Aβ and cognitive deficits [61] and cardiac arrest can rapidly and massively upregulate plasma Aβ levels [62]. Hypoxia has also shown to up-regulate the genes required for Aβ production [63] [64] and here we have shown it to up-regulate PS2V in guinea pig brain. Whether cholesterol up-regulates PS2V, Aβ and Aβ generating genes via impacting on cerebrovascualture, promoting ischemia or hypoxia could not be determined from our data, but could be addressed in *in vitro* or *in vivo* follow up studies by assessing vasculature/hypoxic markers under cholesterol loading conditions. Our data indicate that guinea pigs represent the best *in vivo* model for dissecting the contribution of cholesterol to up-regulation of PS2V and Aβ.

### Limitations of the Guinea Pig as a Model of AD

Despite the overall advantages over other rodent models, there are limitations to the guinea pig in modelling all aspects of AD pathology including neurofibrillary tangles. There is a distinct lack of knowledge of the tau (*MAPT*) isoforms that exist and whether they are hyperphosphorylated. In our attempts to identify *MAPT* transcripts in guinea pig brain, a predicted sequence was obtained through sequence alignments using Guinea pig ESTs and the Ensembl database. This predicted sequence showed a similar degree of identity to human *MAPT* and thus RT-PCR using human primers was used to identify 3R and 4R repeats of *MAPT*. However, isoforms possessing only one amino terminal insert (1N) were identified while up to three isoforms at this site have been found in human MAPT transcripts (0N, 1N and 2N). Only one other study has investigated tau isoforms in guinea pig brain tissue. That study used an antibody against human tau and only detected the 1N isoform [65]. This supports the result of our RT-PCR analysis in which we could only identify 1N transcripts. The Takuma et al [65] study also identified differences in amino-terminal inserts between mice and rats where 1N and 2N insert types are dominant in rats, whilst 0N and 1N is dominant in mice. The reasons for these species differences in amino-terminal isoforms (and indeed their function) remain unclear. However the dominance of the 1N isoform in human, mice, rats and guinea pigs suggest a conserved role for tau containing this particular N-terminal insert.

Despite the lack of all MAPT isoforms, we show that both 3R and 4R MAPT transcript is present in guinea pig brain and that the 3R/4R ratio was altered due to increases in the 3R transcript. Disturbance of the ratio of 4R to 3R is a feature of AD and neurodegenerative tauopathies. The altered ratio is thought to be due to increases in 4R or reductions in 3R tau levels [66], [67]. However, increased levels of 3R tau have been reported to play a role in the progression of tau pathology particularly at mild-to moderate stages of disease severity [68]; [69]. Further, increases in 3R tau, but not 4R tau were reported in brains of aged obese rats that model the AD risk factor, type-2 diabetes, resulting in increased intracytoplasmic aggregates (that were reactive with antibodies against 3R) and synaptic degeneration [70]. We have shown that another AD risk factor, cholesterol intake, increases 3R transcripts, and although to be directly assessed, would most likely result in increased protein levels.

Taken together with the guinea pig’s established role as a model of human lipoprotein and cholesterol metabolism, our findings provide further evidence that they are an alternative *in vivo* model to mice and rats for studying the effects of AD risk factors such as cholesterol on Aβ metabolism and PS2V generation and for evaluating dietary interventions that may have beneficial outcomes in AD.

## Supporting Information

Figure S1 Average animal weights (grams) (A) and Average food consumption (grams/day) (B) between the cholesterol and control diet groups over the 12 week experimental diet. Values mean ± SEM.
(TIF)

Table S1 Dietary composition for the control and the cholesterol diet groups. ^a^The oil mix contained 49% Copha (solidified coconut oil), 27% safflower oil, and 24% olive oil, and was high in lauric and myristic acids known to cause endogenous hypercholesterolemia in guinea pigs. ^b^Mineral and vitamin mixes (AIN_93_G) were formulated to meet the daily requirements for guinea pigs.
(DOCX)
