# Supplementary figures and images for: The Guinea Pig as a Model for Sporadic Alzheimer’s Disease (AD): The Impact of Cholesterol Intake on Expression of AD-Related Genes
Source: PLoS One. 2013 Jun 21;8(6):e66235. doi: 10.1371/journal.pone.0066235 (PMC3689723; doi:10.1371/journal.pone.0066235)

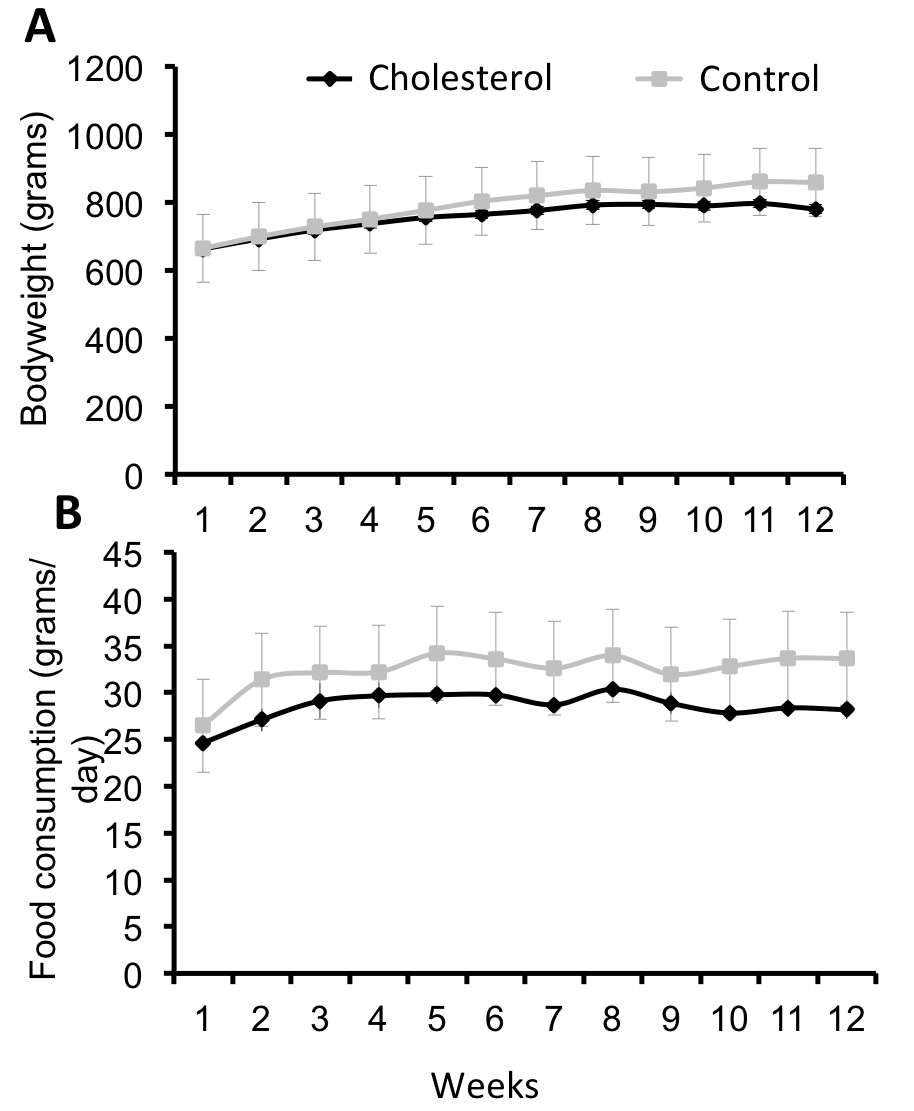

Supplement: Figure S1 — Average animal weights (grams) (A) and Average food consumption (grams/day) (B) between the cholesterol and control diet groups over the 12 week experimental diet. Values mean ± SEM. (TIF) [file pone.0066235.s001.tif]
