# Supplementary material for: The Guinea Pig as a Model for Sporadic Alzheimer’s Disease (AD): The Impact of Cholesterol Intake on Expression of AD-Related Genes
Source: PLoS One. 2013 Jun 21;8(6):e66235. doi: 10.1371/journal.pone.0066235 (PMC3689723; doi:10.1371/journal.pone.0066235)
Supplement: Table S1 — Dietary composition for the control and the cholesterol diet groups. aThe oil mix contained 49% Copha (solidified coconut oil), 27% safflower oil, and 24% olive oil, and was high in lauric and myristic acids known to cause endogenous hypercholesterolemia in guinea pigs. bMineral and vitamin mixes (AIN_93_G) were formulated to meet the daily requirements for guinea pigs. (DOCX) [file pone.0066235.s002.docx]

|  | Control | High-Cholesterol |
| --- | --- | --- |
| Ingredient (g/100g diet) |  |  |
| Sucrose | 13.3 | 13.3 |
| Casein | 13.0 | 13.0 |
| Soy Protein | 8.69 | 8.69 |
| Oil Mix ^a^ | 15.092 | 15.092 |
| Cellulose | 10.90 | 10.9 |
| Guar Gum | 2.71 | 2.71 |
| Wheat Starch | 22.562 | 22.562 |
| Dextrinised Starch | 4.60 | 4.60 |
| DL-Methionine | 0.540 | 0.540 |
| Lime (Fine Calcium Carbonate) | 2.40 | 2.40 |
| Salt (Fine Sodium Chloride) | 0.260 | 0.260 |
| Potassium Dihydrogen Phosphate | 1.30 | 1.30 |
| Potassium Sulphate | 0.160 | 0.160 |
| Potassium Citrate | 0.250 | 0.250 |
| Magnesium Oxide | 0.150 | 0.150 |
| Blue Food Colouring | 0.04 | - |
| Red Food Colouring | - | 0.04 |
| AIN_93_G_Trace Minerals ^a^ | 0.140 | 0.140 |
| AIN_93_G_Vitamins ^a^ | 1.00 | 1.00 |
| Choline Chloride 60% w/w | 0.50 | 0.50 |
| STAY Vit C (35% Vit C) | 1.150 | 1.150 |
| Guinea Pig supplement | 0.999 | 0.999 |
| Cholesterol (USP) | 0.04 | 0.250 |
|  |  |  |
| Protein (% energy) | 25 | 25 |
| Carbohydrate (% energy) | 41 | 41 |
| Fat (% energy) | 34 | 34 |
|  |  |  |
| Digestible Energy | 16.1 MJ/Kg | 16.1 MJ/Kg |
